# Assessing the Safety of Semaglutide and Tirzepatide in Black and Asian Populations: A Narrative Review

**DOI:** 10.7759/cureus.87188

**Published:** 2025-07-02

**Authors:** Pulwasha Iftikhar, Chander P Khatri, Sri Ratna Divya Nanduri, Minahil Ramzan, Krishna Sai Kiran Sakalabaktula

**Affiliations:** 1 Obstetrics and Gynecology, City University of New York, New York, USA; 2 Internal Medicine, Marshall University, Huntington, USA; 3 Internal Medicine, St. Joseph's Medical Center, Stockton, USA; 4 Internal Medicine, Corewell Health Hospital, Dearborn, USA; 5 Biomedical Informatics and Genomics, Tulane University, New Orleans, USA

**Keywords:** asian people, black population, glp-1 agonist, racial disparities, safety profile, semaglutide, side effects, tirzepatide

## Abstract

Semaglutide and tirzepatide represent new treatment modalities for type 2 diabetes mellitus (T2DM) and obesity, expanding the range of glucagon-like peptide-1 (GLP-1) receptor agonists. Semaglutide is a GLP-1 receptor analog while tirzepatide is a dual agonist that targets both glucose-dependent insulinotropic polypeptide (GIP) and GLP-1 receptors. Both are effective and well-tolerated treatments for managing T2DM and obesity, but their safety and effectiveness may vary across racial and ethnic groups. South Asians and Black individuals experience disproportionately higher rates of both obesity and diabetes compared to other racial and ethnic groups. These health disparities, along with socioeconomic barriers, highlight the urgent need for research to explore how semaglutide and tirzepatide perform across diverse populations.

Asian populations, such as Japanese patients, appear to experience higher rates of gastrointestinal side effects with GLP-1 receptor agonists like semaglutide and tirzepatide compared to other groups. Additionally, South Asian adults who are older and have a lower BMI are more likely to discontinue the medication due to the heightened impact of side effects, particularly weight loss. Furthermore, Black populations also experience significant cardiovascular benefits with GLP-1 analogs, although the differences are less pronounced than in Asians.

To ensure the best results, treatment plans should be tailored to the specific needs of individual patients, considering their ethnic backgrounds and socio-economic conditions, among other factors. This can be reinforced by improving patient education, offering support programs, and implementing policy changes to enhance medication adherence across diverse populations. Further research is needed to understand better how these factors vary across different populations and to optimize treatment strategies accordingly.

## Introduction and background

Semaglutide and tirzepatide represent new treatment modalities in type 2 diabetes mellitus (T2DM) and obesity and increase the range of glucagon-like peptide-1 (GLP-1) receptor agonists (GLP-1RAs). Semaglutide is a GLP-1 analog; since 2017, it has been highly effective in glucose control and weight management [1]. In clinical trials, semaglutide has been shown to significantly reduce hemoglobin A1C (HbA1C) levels by 1.5% to 1.9% and decrease body weight by up to 15%, positioning it as a strong option for managing T2DM [1,2]. Tirzepatide, a newer agent, received FDA approval in 2022 as Mounjaro for T2DM and was approved in 2023 for obesity [3]. This dual GLP-1 and glucose-dependent insulinotropic polypeptide (GIP) receptor agonist helps in managing both T2DM and obesity by leveraging its dual-action mechanism, which has been associated with improvements in weight reduction and glycemic control [4]. Both medications are also linked to favorable cardiovascular outcomes, reflecting their potential to impact broader health aspects in T2DM patients [1]. Moreover, gastrointestinal adverse effects, including nausea, constipation, and diarrhea, remain a common issue with these drugs [5].

**Figure 1:**
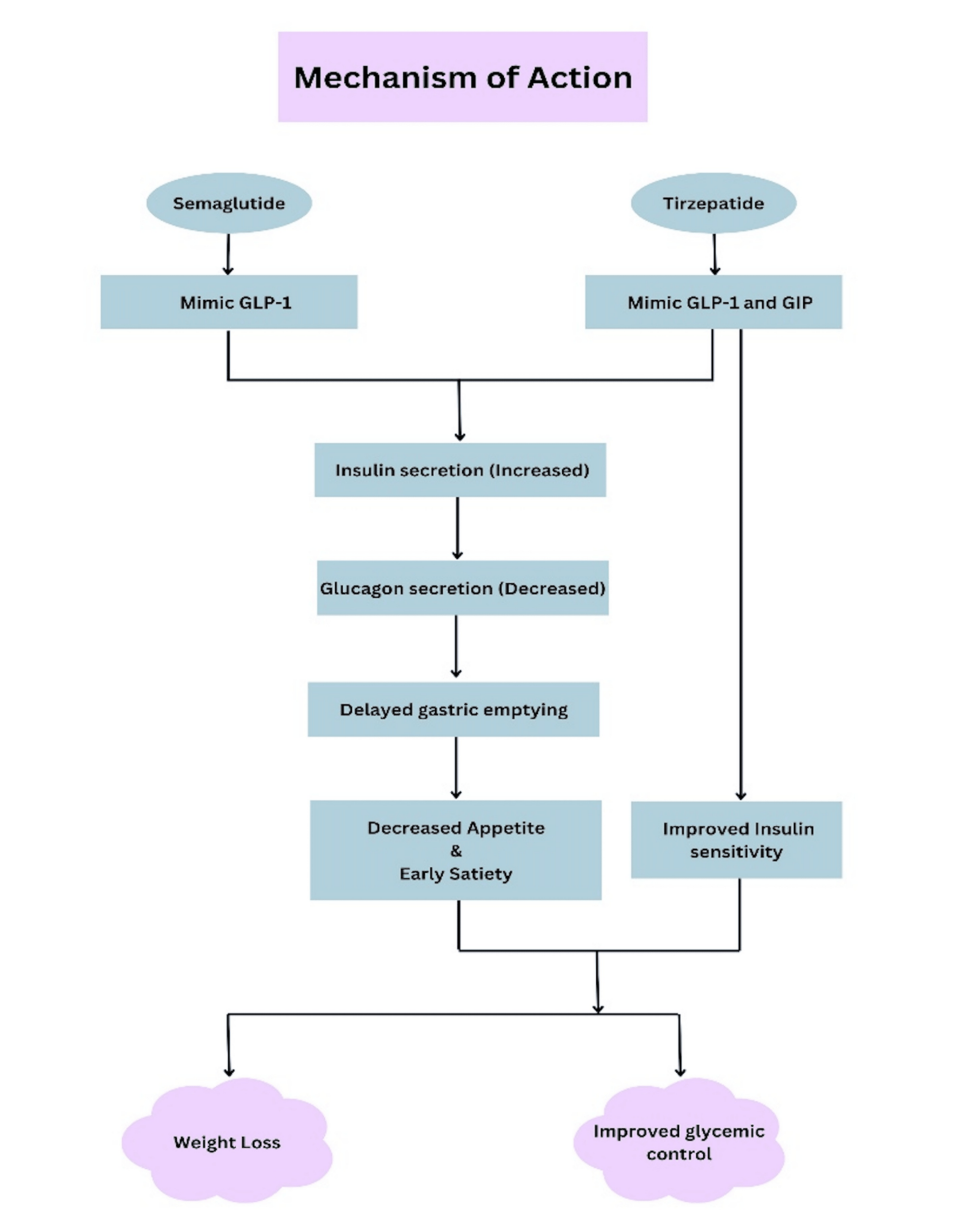
Flow Chart Depicting Mechanism of Action of Semaglutide and Tirzepatide Created by the author using Canva GLP-1: glucagon-like peptide-1, GIP: glucose-dependent insulinotropic polypeptide

The journey of GLP-1RAs began with the approval of exenatide in 2005, the first medication of its kind. Since then, several other medications have joined the lineup, including liraglutide, albiglutide, and dulaglutide [6]. Semaglutide took things a step further by offering both injectable and oral forms, and it provided significantly greater BMI reduction compared to older GLP-1 receptor agonists like exenatide and liraglutide. The introduction of the oral version in 2019 was a notable breakthrough, as it was the first GLP-1RA that didn’t require injections for managing T2DM [7]. More recently, tirzepatide has made waves with its dual action on GIP and GLP-1 receptors. Like semaglutide, it’s designed for once-weekly injections and has a convenient half-life of about five days. This means it doesn’t need to be taken daily, which is convenient for patients [7]. GLP-1RAs, including semaglutide and tirzepatide, work in several ways to help manage blood sugar and support weight loss [8]. They enhance insulin secretion, reduce glucagon release, promote satiety, and slow gastric emptying. Together, these effects help keep blood glucose levels in check and support weight management [6]. Figure 1 describes the mechanism of action of semaglutide and tirzepatide and their way of promoting weight loss and improving glycemic control.

Similarly, the obesity epidemic is predicted to almost double in Southeast Asia by 2030 [9]. Furthermore, in the United States, African American populations show higher rates of obesity and diabetes compared to other racial groups [10]. The disparities in health outcomes among these groups emphasize the need for focused research to understand how semaglutide and tirzepatide perform across different ethnicities. This includes examining potential variations in efficacy and safety profiles that may impact treatment outcomes and adherence.

Understanding how GLP-1 receptor agonists work in different populations is key to fine-tuning treatment strategies. For example, people in East Asia, including Southeast Asia, often have lower levels of GLP-1 due to differences in how their bodies produce and break down this hormone [9]. This might mean they could benefit more from GLP-1RAs, which could be a valuable tool in managing blood sugar levels effectively. Similarly, socioeconomic factors and healthcare access can limit how effectively Black adults benefit from medications like semaglutide. Barriers such as high out-of-pocket costs or lack of insurance coverage can make these treatments financially inaccessible to those with limited resources, even though the medication may be highly effective when accessible [10]. These disparities can affect not only treatment outcomes but also treatment adherence plans. Therefore, this literature review aims to evaluate the safety and efficacy of semaglutide and tirzepatide specifically in Asian and Black populations, to bridge existing research gaps, advance personalized treatment approaches, and ensure all patients have the best possible outcomes and access to equitable healthcare.

## Review

Methods

We chose PubMed as our primary database for this research due to its comprehensive collection of biomedical literature. Additionally, we used Cochrane, Google Scholar, and Web of Science to identify relevant articles. We focused our search using key terms such as 'GLP-1 receptor agonist,' 'GIP receptor agonist,' 'semaglutide,' 'tirzepatide,' 'safety profile,' 'side effects,' 'racial disparity', 'Asians,' and 'Black population.' To ensure the comprehensiveness of the data, two reviewers independently assessed the available articles, including only those published in English.

Pharmacokinetics and Pharmacodynamics

Medication responses can vary widely among different ethnic groups due to differences in how drugs are processed in the body [11]. For South Asians, unique factors such as variations in body fat distribution and specific genetic traits can influence how medications like tirzepatide and semaglutide are absorbed, distributed, metabolized, and eliminated. These differences may impact the effectiveness of these drugs for Asians, though research is still limited. However, existing studies suggest that, despite these variations, the overall safety profiles of tirzepatide and semaglutide for South Asians are generally consistent with those observed in other populations [12,13]. Similarly, in African American populations, genetic differences, especially those related to CYP450 enzymes, can affect how drugs are metabolized [14]. These genetic variations might lead to different drug efficacy and side effects in patients taking tirzepatide or semaglutide. Understanding these differences is crucial for tailoring treatments and improving outcomes across diverse populations.

Safety Profile of Tirzepatide and Semaglutide in Type 2 Diabetes Mellitus

Recent research has reinforced that tirzepatide is effective and safe for managing T2DM, including across diverse populations. A study by Feng et al. showed that the once-weekly subcutaneous injection of tirzepatide was generally well-tolerated by Chinese patients [15]. The most common side effects were mild and mostly related to the digestive system, like diarrhea and a reduced appetite. These issues were self-limiting and didn’t require medical treatment. This study’s findings are consistent with the broader SURPASS clinical trial program, which also reported a low rate of hypoglycemia, further supporting tirzepatide’s safety [15,16]. Moreover, no serious adverse events were recorded in the tirzepatide group, although there was a single case of acute myocardial infarction in the placebo group. Overall, these results underscore tirzepatide’s favorable safety profile, particularly in Asian ethnic groups.

**Figure 2:**
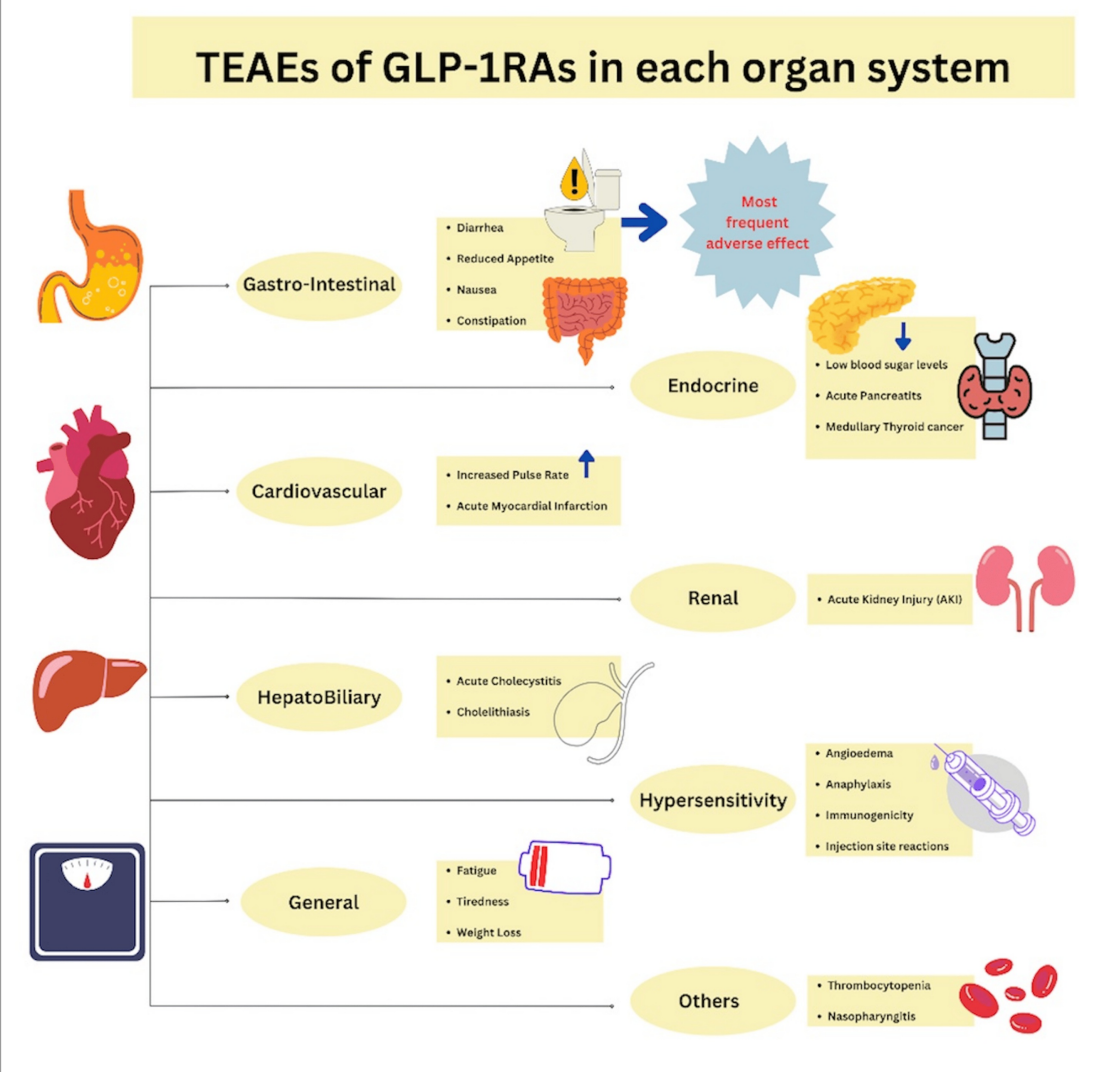
Treatment Emergent Adverse Effects (TEAEs) of Glucagon-Like Peptide-1 Receptor Agonists (GLP-1RAs) in Each Organ System Created by the author using Canva

In 2022, Kiyosue et al. analyzed the safety and efficacy of tirzepatide in East Asian individuals with type 2 diabetes as part of the SURPASS program [16]. The study found that 70.1% to 86.9% of participants experienced treatment-emergent adverse events (TEAEs), mainly gastrointestinal (GI), with serious events in 0% to 12.8%. Figure 2 demonstrates the TEAEs of GLP-1RAs in each organ system. Discontinuation rates due to adverse effects were highest in patients with a BMI under 25 kg/m² and those over 65, though overall adverse event rates were not higher in these groups. The post hoc analysis in this study highlighted that tirzepatide was well tolerated and had a favorable benefit/risk profile in East Asian participants over a range of baseline BMI values and ages, and suggested that lower doses may be beneficial for older patients and those with lower BMI, underscoring the need for personalized treatment. 

A systematic review and meta-analysis by Cui et al. assessed the comparative efficacy and safety of tirzepatide in Asians and Non-Asians with type 2 diabetes. This study found a significant dose-dependent relationship between tirzepatide dosage and GI adverse events, evaluating six types of adverse effects across 5 mg, 10 mg, and 15 mg doses. Asian populations tend to have higher body fat percentages and greater visceral adiposity compared to non-Asian groups. They were more prone to weight loss and GI side effects. In contrast, non-Asian individuals tended to achieve better blood sugar control but experienced a higher incidence of metabolic and nutritional imbalances. The analysis emphasized the need for dose adjustments of tirzepatide based on racial differences, despite its limitations [12]. 

Metabolic and Cardiovascular Effects With Long-Acting GLP-1 Receptor Agonists

Recent clinical trials and studies have provided insights into the efficacy and safety of tirzepatide, a novel GLP-1 receptor agonist, in various Asian populations. In a Phase 3 trial with Japanese participants, tirzepatide was compared to dulaglutide and showed a safety profile similar to other GLP-1 receptor agonists. However, it did come with some common GI side effects, such as nausea, constipation, and nasopharyngitis, which were reported more frequently than with dulaglutide. About 10% of participants in the 10 mg and 15 mg tirzepatide groups had to stop the treatment due to these adverse effects. Despite these challenges, tirzepatide proved more effective at reducing HbA1c levels than seen in the global SURPASS studies. This suggests that individual factors, such as body weight and physiological differences among Japanese patients, might influence the treatment response [17].

A detailed assessment of tirzepatide’s tolerability and safety in a Japanese cohort, which included 443 participants, revealed that the frequency of TEAE was notably higher in the 15 mg group compared to the 5 mg and 10 mg groups. Common TEAEs included nasopharyngitis, nausea, constipation, and diarrhea, with a higher incidence of nasopharyngitis observed in this study compared to global trials. The increased pulse rate associated with tirzepatide was more pronounced in this cohort than in previous global studies, although its clinical significance remains uncertain. Overall, the safety profile of tirzepatide in Japanese patients aligns with global findings, confirming its general tolerability as an add-on therapy to oral antihyperglycemic medications [18].

Moreover, a Phase 3 trial conducted with 917 patients from China, South Korea, Australia, and India compared tirzepatide with insulin glargine. Tirzepatide was generally well tolerated, with GI issues such as decreased appetite, nausea, and diarrhea being the most common adverse events. Treatment discontinuations due to adverse effects were higher in the 10 mg and 15 mg tirzepatide groups compared to the 5 mg group and insulin glargine. Importantly, no serious adverse effects were reported, indicating a favorable safety profile. Tirzepatide demonstrated superior efficacy in reducing HbA1c levels compared to insulin glargine, highlighting its potential as an effective treatment option for T2DM in Asian populations [19].

The findings from these studies highlight tirzepatide’s effectiveness and safety across various Asian populations, although they also reveal some differences in side effects and treatment responses. For instance, Japanese patients experienced a higher rate of GI issues compared to other Asian sub-groups, which suggests the need for tailored treatment approaches. Despite these variations, tirzepatide stands out as a promising option for managing blood sugar levels and supporting weight loss in people with T2DM. Its safety profile is on par with other GLP-1 receptor agonists [17-19]. However, more research is needed to fully understand how tirzepatide performs in different geographic regions and among different populations in its efficacy and tolerability to ensure the best possible outcomes for all patients.

Moreover, Joseph et al. conducted a thorough review of the cardiovascular impacts of race and ethnicity in patients with diabetes and obesity, analyzing data from 3,074 subjects in the SUSTAIN 1-5 and 7 trials, and 1,648 subjects in the SUSTAIN 6 trial. The findings highlighted differing responses to GLP-1 agonists, such as semaglutide and liraglutide, among Asian, Hispanic, and Black populations. For example, the semaglutide SUSTAIN 6 trial reported hazard ratios for major adverse cardiovascular events of 0.58 in Asians and 0.72 in Black people. The review noted lower prescription rates of GLP-1 agonists and SGLT-2 inhibitors among racial and ethnic minority groups in the U.S., emphasizing the need for patient-centered, guideline-based care for these underserved populations [20].

Discussion

This literature review evaluated the safety profiles of semaglutide and tirzepatide among Southeast Asian and Black populations, focusing on their efficacy in managing T2DM and obesity. Both medications have shown significant benefits in lowering HbA1c levels and body weight. However, understanding the safety profile across different racial and ethnic groups is essential. This is particularly important given the observed variations in different groups' treatment responses and the frequency of adverse events. By exploring these differences, we can better tailor treatments to ensure they are effective and safe for diverse populations.

Tirzepatide, a dual GLP-1 and GIP receptor agonist, has shown efficacy in managing T2DM and obesity. The SURPASS clinical trial program demonstrated effectiveness in improving glycemic control and promoting weight loss. However, GI adverse effects, such as nausea and diarrhea, are commonly reported. In Southeast Asian populations, particularly among Chinese individuals, these GI issues are prevalent but generally mild and self-limiting [21]. Research indicates that while GI events are consistent with global findings, the frequency and severity can vary based on the dosage of the medication and population-specific factors. A systematic review highlighted a dose-dependent increase in GI adverse events among Asians compared to non-Asians, suggesting that lower doses might be preferable for minimizing these effects [12]. Data specific to populations is less extensive, but general observations from clinical trials indicate that the safety profile of tirzepatide aligns with global safety data. The increased prevalence of metabolic and cardiovascular conditions in Black populations might affect their response to treatment and management of adverse effects [9]. While no new safety concerns specific to Black individuals have emerged, further studies are necessary to understand their response to tirzepatide more comprehensively [9].

**Figure 3:**
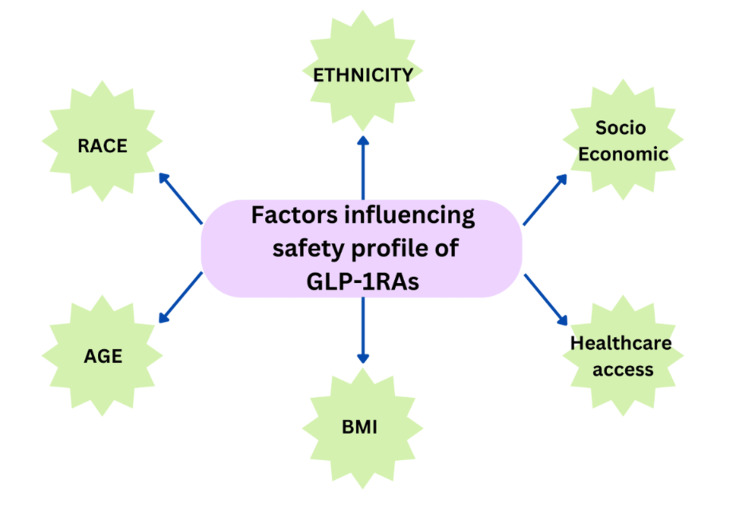
Factors Influencing the Safety Profile of Glucagon-Like Peptide-1 Receptor Agonists (GLP-1RAs) Created by the author using Canva

Semaglutide is well-known for its effectiveness in lowering HbA1c levels and supporting weight loss. However, it has some common side effects, particularly GI side effects such as nausea and diarrhea. Studies from Japan have found that these side effects are more frequent with semaglutide compared to medications like sitagliptin [22]. While these findings are consistent with the global observations, they highlight the need to potentially adjust dosages or combine treatments to better manage these side effects in Asian populations. Furthermore, older adults and those with a lower BMI are more likely to stop the medication, suggesting that careful patient selection and dose adjustments are important [20,9]. For Black populations, the SUSTAIN trials have shown that semaglutide's effectiveness and safety are generally consistent across different racial and ethnic groups. However, Black individuals often start with higher blood pressure and lower triglyceride levels, which can influence how they respond to the treatment [10]. Socioeconomic factors and differences in healthcare access can also affect treatment outcomes and adherence among Black populations [22,23,9]. Understanding these factors helps in providing more personalized and effective care. Figure 3 describes the factors that could influence the safety profile of GLP-1 receptor agonists. 

The meta-analysis by Kang et al. highlighted that long-acting GLP-1 receptor agonists, such as trizepatide and semaglutide, provide significant cardiovascular benefits in Asian populations [9]. This increased benefit is attributed to the more significant metabolic impact in these groups, along with a reduced risk of major adverse cardiovascular events (MACE) in Asians, likely due to lower baseline GLP-1 levels [9,11]. Additionally, Black populations have also significant cardiovascular advantages with GLP1 analogs but, the differences were less pronounced compared to Asian groups [11]. This suggests that even if GLP-1 receptor agonists are superior in treatment for individuals from various ethnic backgrounds, the extent of cardiovascular benefit can differ, highlighting the need for personalized treatment approaches to optimize therapeutic outcomes and minimize adverse effects.

Chuang et al. and Cobo et al. examined racial disparities in medication adherence and treatment results, reporting that Black and South Asian populations may experience differing outcomes to therapies such as semaglutide and tirzepatide [24,25]. Expanding upon these disparities, Karagiannis et al. highlight how socioeconomic factors and medication adherence affect the effectiveness of GLP-1 receptor agonists like semaglutide and tirzepatide. Factors such as income, education, and access to healthcare significantly influence how well patients follow their medication regimens. Individuals with lower socioeconomic status often encounter significant barriers in accessing medications and healthcare services, which can result in delayed treatment initiation or suboptimal outcomes due to limited financial resources or inadequate insurance coverage [26]. These variations highlight the need for targeted interventions to address socio-economic barriers. The authors suggest improving patient education, offering support programs, and implementing policy changes to enhance medication adherence and treatment success across diverse populations [25-27]. We emphasize the need for future research to thoroughly evaluate the efficacy and safety profiles of semaglutide and tirzepatide across diverse demographic groups and subgroups, particularly among Asian and Black populations. Current data remain limited, and subgroup-specific analyses are often lacking or underrepresented in clinical trials. Addressing these gaps is essential to ensure equitable, evidence-based treatment decisions and to better understand potential variations in drug response and side-effect profiles among different populations.

## Conclusions

Semaglutide and tirzepatide have proven to be effective and well-tolerated treatments for managing T2DM and obesity, showing notable improvements in both blood sugar control and weight loss. However, their safety and effectiveness can vary across different racial and ethnic groups. To ensure the best results, it’s important to tailor treatment plans to the specific needs of individual patients, considering their ethnic backgrounds and socio-economic conditions. Further research is required to better understand these variations. This includes conducting long-term studies to track the ongoing safety and efficacy of these medications, optimizing dosages for different racial groups, and examining how socio-economic factors impact medication adherence and access. By addressing these issues, we can better advance personalized medicine and work towards improving treatment outcomes across diverse populations.
